# Coronaviruses Associated with the Superfamily *Musteloidea*

**DOI:** 10.1128/mBio.02873-20

**Published:** 2021-01-19

**Authors:** Alison E. Stout, Qinghua Guo, Jean K. Millet, Ricardo de Matos, Gary R. Whittaker

**Affiliations:** a Department of Microbiology & Immunology, Cornell University, Ithaca, New York, USA; b Master of Public Health Program, Cornell University, Ithaca, New York, USA; c Université Paris-Saclay, INRAE, UVSQ, Virologie et Immunologie Moléculaires, Jouy-en-Josas, France; d Department of Clinical Sciences, College of Veterinary Medicine, Cornell University, Ithaca, New York, USA; Albert Einstein College of Medicine

**Keywords:** coronavirus, ferret coronavirus, mink, mustelids, *Musteloidea*, SARS-CoV-2

## Abstract

Among the animal superfamily *Musteloidea*, which includes those commonly known as mustelids, naturally occurring and species-specific alphacoronavirus infections have been observed in both mink (Mustela vison*/*Neovison vison) and domestic ferrets (Mustela putorius furo). Ferret systemic coronavirus (FRSCV), in particular, has been associated with a rare but fatal systemic disease. In recent months, it has become apparent that both minks and ferrets are susceptible to severe acute respiratory syndrome coronavirus 2 (SARS-CoV-2), a betacoronavirus and the cause of the coronavirus disease 2019 (COVID-19) pandemic. Several mink farms have experienced SARS-CoV-2 outbreaks, and experimental models have demonstrated susceptibility of ferrets to SARS-CoV-2. The potential for pet ferrets to become infected with SARS-CoV-2, however, remains elusive. During the 2002–2003 SARS epidemic, it was also apparent that ferrets were susceptible to SARS-CoV and could be utilized in vaccine development. From a comparative standpoint, understanding the relationships between different infections and disease pathogenesis in the animal superfamily *Musteloidea* may help elucidate viral infection and transmission mechanisms, as well as treatment and prevention strategies for coronaviruses.

## Abstract (precis)

Among the animal superfamily *Musteloidea*, which includes those commonly known as mustelids, naturally occurring and species-specific alphacoronavirus infections have been observed in both mink (Mustela vison*/*Neovison vison) and domestic ferrets (Mustela putorius furo). Ferret systemic coronavirus (FRSCV), in particular, has been associated with a rare but fatal systemic disease.

## INTRODUCTION

The superfamily *Musteloidea* is comprised of four families: *Ailuridae* (including red pandas), *Mustelidae* (including weasels, otters, and badgers), *Procyonidae* (including raccoons), and *Mephitidae* (including skunks) (1). Across the *Mustelidae* family, domestic ferrets (Mustela putorius furo) are frequently kept as pets or used in various laboratory models of human infectious diseases, including severe acute respiratory syndrome (SARS), influenza, and respiratory syncytial virus (2–4) and American mink (Mustela vison*/*Neovison vison) have been widely raised as fur-bearing animals. Additional species, such as the European mink (Mustela lutreola) and black-footed ferret (Mustela nigripes), face population threats in the wild (5, 6). These unique environments carry different risks for infectious diseases but can have impacts on zoonotic disease spread, conservation efforts, laboratory investigations, and economic potential for farm-raised species. Most recently, it became apparent that ferrets can be experimentally infected with severe acute respiratory syndrome coronavirus 2 (SARS-CoV-2), as with SARS-CoV (4, 7–9). Natural infection by SARS-CoV-2, from human to mink, has also been reported on mink farms (10–12), as well as spill-back to humans (13). In addition to infection with these human coronaviruses, several species-specific coronaviruses have been described in *Mustelidae*, including ferret enteric coronavirus (FRECV), ferret systemic coronavirus (FRSCV), and epizootic catarrhal gastroenteritis in mink (14–16). Less is known in regard to coronavirus infections in the other *Musteloidea* families but must be considered in our understanding of disease transmission.

FRECV, FRSCV, and mink coronaviruses (MCoVs) belong to the alphacoronavirus genus within the family *Coronaviridae* (15, 17). SARS-CoV and SARS-CoV-2 belong to the betacoronavirus genus, along with the Middle East respiratory syndrome coronavirus (MERS-CoV) and several other human coronaviruses (18). The *Coronaviridae* are single-stranded, positive-sense RNA viruses with relatively large genomes, approximately 32 kb (19). The mutation rate in coronaviruses is relatively high, on the order of 10^−4^ nucleotide substitutions/site/year, despite RdRp proofreading, exoribonuclease activity of nonstructural protein 14 (nsp 14) (20). Along with the known propensity for recombination, the ability of coronaviruses to undergo mutation has aided in outbreaks of coronavirus disease in novel hosts (21). Novel and emerging viruses thus may create significant risks across the environments that *Mustelidae* species occupy (22). Here, we review the emergence of coronaviruses in *Musteloidea* species.

## MINK

In 1975, Larsen and Gorham reported on a novel disease causing enteritis, as well as anorexia and mucoid diarrhea in mink in the United States, especially during the fall season and in darker colored mink (14), terming this disease epizootic catarrhal gastroenteritis. Over a decade later, electron microscopy and transmission experiments implicated a coronavirus as the cause of epizootic catarrhal gastroenteritis (23). Further serological study of Danish mink showed cross-reactivity with transmissible gastroenteritis virus (TGEV) of pigs (24). More recently, phylogenetic analysis of two novel mink coronaviruses (MCoVs) grouped these isolates within the alphacoronavirus genus, with similarities to the ferret coronaviruses (17). The inability to isolate either one of the two MCoVs in cell culture, more recently, may have been due to low sample quality or lack of viable CoV after sample storage (17). The most recent surveillance for epizootic catarrhal gastroenteritis in farmed mink (Mustela vison*/*Neovison vison) in North America demonstrated that 14 of 339 (4.1%) animals submitted for necropsy were afflicted, with the disease most common in juvenile mink (25).

Following the SARS outbreak of 2003, interest in mink was sparked when it was demonstrated that mink lung cells (Mv 1 Lu) were permissive to SARS-CoV infection (26–28) and express angiotensin-converting enzyme 2 (ACE2), the SARS-CoV and SARS-CoV-2 receptor (29–31). The coronavirus disease 2019 (COVID-19) pandemic has additionally drawn attention to coronavirus epidemiology and pathogenesis in mink, with several fur farms, in the United States, Netherlands, Denmark, and Spain, having associated SARS-CoV-2 outbreaks, presumably introduced via farm personnel (10, 12, 13). Respiratory signs, considered severe in some cases, as well as death were observed in animals across the outbreak locations (11, 12). Interstitial pneumonia was present in numerous animals that succumbed to viral infection (12).

## FERRETS

**FIG 1:**
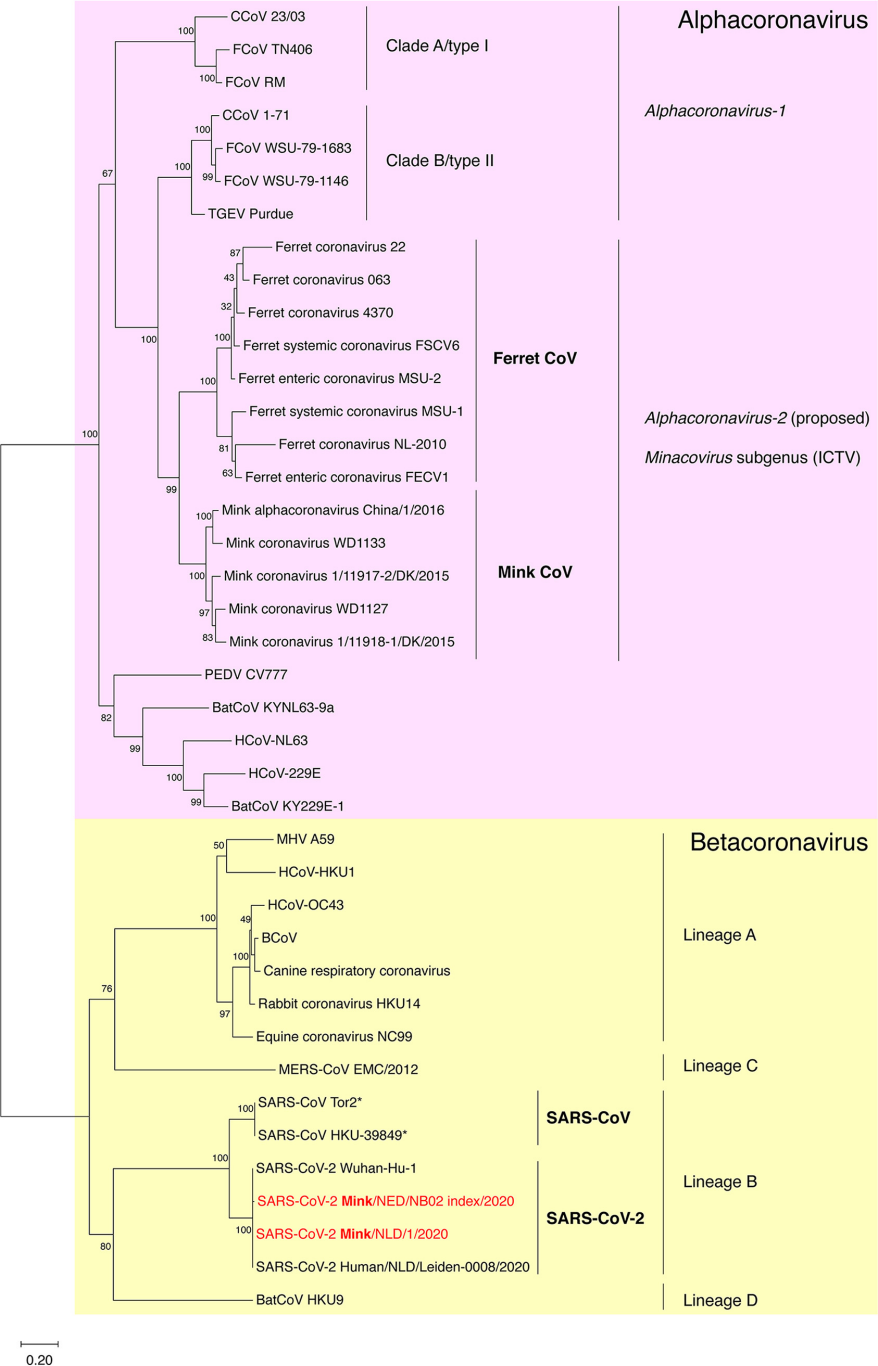
Coronaviruses of minks and ferrets. Phylogenetic analysis based on the spike protein sequences of representative alpha- and betacoronaviruses, including viruses infecting mustelids. A maximum likelihood (ML) phylogenetic tree was generated based on selected spike protein sequences. Bootstrap values shown at nodes were calculated from 1,000 replicates. The tree is drawn to scale, with branch lengths measured in the number of substitutions per site. SARS-CoV-2 strains in red font denote isolates from minks. Strains of SARS-CoV that were used to experimentally infect ferrets are indicated by an asterisk.

In 1993, the diarrheal disease epizootic catarrhal enteritis (ECE) was first noted in domestic ferrets (Mustela putorius furo) in the United States (16). The common clinical feature associated with ECE is “profuse, bright green diarrhea,” though other common signs include vomiting, nonspecific lethargy, and inappetence, with disease particularly affecting older animals compared to younger animals (16). However, the potential for asymptomatic carriage remains evident (32). In the initial report of ECE, viral isolation was attempted but was unsuccessful, although electron microscopy identified a coronavirus in the feces and jejunum of animals with clinical disease (16). The causative agent of ECE is ferret enteric coronavirus (FRECV) and molecular characterization grouped this virus within the genus alphacoronavirus, with similarity to TGEV, feline coronavirus (FCoV) and canine coronavirus (CCoV) (33). Additional phylogenetic analysis revealed the close relationship between ferret coronaviruses (FRCoV) with mink coronaviruses (Fig. 1), as previously suggested, based on ORF1ab and full genome phylogeny (34, 35). Together, ferret and mink coronaviruses comprise a distinct species, previously proposed as *Alphacoronavirus-2* (Fig. 1), though not currently in use by the International Committee on Taxonomy of Viruses (ICTV). Instead, mink coronavirus 1 is currently classified by ICTV as subgenus *Minacovirus*, along with ferret coronavirus (18) (Fig. 1). In two strains reported by Minami et al., phylogenetic analysis has indicated potential recombination events leading to the emergence of two additional strains, Saitama-1 and Aichi-1 (35). Lamers and colleagues have similarly shown recombination events among the S, 3c, and E genes of the FRCoVs through comparison of a strain identified in the Netherlands, designated FRCoV-NL-2010, to the previously described FRSCV MSU-1 and FRECV MSU-2 (34). Most recently, our lab has reported on systemic disease, including bone marrow involvement, in a ferret infected with virus closely related with FRECV-MSU-2 based on the spike sequence (36).

A second disease-causing coronavirus, ferret systemic coronavirus (FRSCV), was first described in 2004 in ferrets in Spain (15) and phylogenetically closely related to FRECV (37). In this initial case series, it became apparent that infection with FRSCV could result in systemic disease similar to feline infectious peritonitis (FIP), an invariably fatal disease of cats that occurs in rare cases upon infection with FCoV (15, 38). Bloodwork results on these ferrets revealed anemia and hypergammaglobulinemia and immunohistochemical staining demonstrated viral antigen across numerous tissues: lung, liver, kidney, spleen, lymph nodes, intestine, heart, and pancreas (15). Variable granulomatous lesions were present on histology (15). Shortly after the initial report of FRSCV, additional cases have been described, with a wide range of clinical signs, including diarrhea, hind limb weakness, inappetence, cluster seizures, and other neurological abnormalities, icterus, palpation of abdominal masses, organomegaly, coughing, vomiting, bruxism, rectal prolapse, panophthalmitis, systolic murmur, and skin erythema (39–44). Common findings on complete blood count (CBC) panels include anemia, (neutrophilic) leukocytosis, and thrombocytopenia (39–41, 43, 45–47). Common chemistry panel aberrations have included hyperproteinemia, hypoalbuminemia, hypergammaglobulinemia, azotemia, increased alanine aminotransferase (ALT), increased serum lipase, and variable other disturbances (39, 40, 43, 47).

Infections have encompassed numerous scenarios, including both pet ferrets and laboratory ferrets. In a study of 63 laboratory ferrets, fecal samples from 60 individuals were positive for one or more ferret coronaviruses via reverse transcription-PCR (RT-PCR) in apparently healthy animals (48). Of these samples, 38 fecal samples were positive for FRECV only, 7 were positive for FRSCV only, and 15 fecal samples were positive for both viruses (48). In a separate study of 39 ferrets, 5 of 12 ferrets from a farm in the Netherlands, 4 ferrets from a farm in Sweden, 4 of 12 healthy pet ferrets, and 1 of 3 pet ferrets with diarrhea were positive for FRCoV RNA, based on RT-PCR (49). Comparatively, in Japanese ferrets presenting to animal hospitals, over half of presenting ferrets were positive via RT-PCR (44 of 79 in one study and 126 of 201 in another) (35, 50). In the initial 2014 study, 25 of 34 ferrets with diarrhea were shedding FRCoV, while 17 of 33 animals classified as asymptomatic/nonrelated signs were shedding FRCoV, but genotyping of the viruses failed to show an association between disease and what were classified as genotype I samples versus genotype II samples (50). In the 2016 study, samples from ferrets under 1 year of age were commonly positive (31/38), especially compared to those over 3 years of age (61/110) (35). However, in those ferrets 3 years of age or older, 77.3% of those with diarrhea were positive for virus compared with 40.9% of those without diarrhea (35). In regards to sex, 60.7% of males were positive and 64.9% of females were positive (51). The presence of FRCoV, however, was associated with diarrheal disease in a study by Minami and colleagues (35). Minami and colleagues previously established an enzyme-linked immunosorbent assay (ELISA) to assess seroprevalence; in the validation of their assay, 31 of 35 ferrets were determined to have antibodies against FRCoV (51). Interestingly, age was not significantly associated with seroprevalence but followed an almost oscillating pattern: all ferrets under 1 year of age were seropositive, which then dropped to 67% seroprevalence in 1-year-old ferrets, returned to 100% seroprevalence in 3-year-old ferrets, and then again dropped to 88% seroprevalence in those older than 3 years old (51). Seroprevalence was not associated with sex (51). However, the sex predilection for developing systemic disease due to FRSCV remains understudied. For example, in a study of ferrets that developed systemic disease, 18 of 23 included animals were neutered males, which may represent a biological difference, similar to COVID-19, or was simply a result of sampling method or other nonrelevant factors (40).

Like FIP, FRSCV-associated disease can be challenging to diagnose (52). The monoclonal antibody FIPV 3-70 is the gold standard FIP diagnosis using immunohistochemistry, and it has previously been used in FRSCV diagnostics (15). FIPV 3-70 is targeted to the conserved nucleocapsid (N), and it had been proposed by the antibody manufacturer to recognize SARS-CoV-2 N. As such, there is concern with using this antibody based on the potential for cross-reaction with SARS-CoV-2 and the inability to distinguish between the two viruses, especially if used at low dilutions. Preliminary studies in our laboratory suggest cross-reactivity across the alpha- and betacoronaviruses is not a concern at the dilutions typically used in veterinary pathology services. However, care should clearly be taken in interpreting immunohistochemistry results using this antibody, and efforts made to improve diagnostics using more specific RNA-based *in situ* hybridization techniques. In 2016, Minami and colleagues developed an ELISA test to assess FRCoV antibody, specifically based on the N gene (51). Dominguez and colleagues have previously reported common imaging findings in ferrets with FRSCV (45).

Like other diagnostic options for FRSCV, ultrasonographic and radiographic findings are often nonspecific and support a diagnosis but do not confirm a diagnosis of FRSCV. In general, radiographic findings include lumbar musculature losses (8/8), loss of abdominal serosal detail (7/8), decrease in abdominal contrast (3/8), a pendulous abdomen (3/8), gastrointestinal dilation (3/8), splenomegaly (4/8), abdominal masses (5/8), and nephromegaly (2/8) (45). Ultrasonographic findings support the presence of peritonitis in addition to splenomegaly, lymphadenopathy and changes to lymph node echogenicity, presence of abdominal masses, and variable kidney changes (45).

Postmortem, lesions similar to FIP are described in FRSCV and have been classified by some authors into four patterns: “granulomas without necrosis (G), granulomas with necrosis (G-N), granulomas with neutrophils (G-NL), and diffuse granulomatous inflammation (DG)” (53), similarly described by Martínez et al. (15). As with cats, the distribution of these lesions can vary across individual animals and organs (15, 53, 54). However, unlike FIP, or even COVID-19, FRSCV disease appears less likely to result in the development of vasculitis (53, 55). Despite this, plasma cells have been noted to be perivascular, and necrotizing vasculitis in a lymph node has been observed, with inflammation of the “adventitial and medial tunics of small veins and venules” also described (15, 40, 56). Further investigation into the development, or lack thereof, of vasculitis and related pathology, following FRSCV may help provide comparative insight into systemic coronavirus diseases.

To date, no FDA-approved, specific treatment method exists for FRSCV. Described treatment methods have included steroids, antimicrobials, sucralfate, fluids, and nutritional support but have not resulted in disease resolution (39, 46, 47). Most recently, a 3C-like protease was previously expressed to assess inhibition by several protease inhibitors (57). *In vitro*, the effects of protease inhibitors targeting the 3C-like protease appears to be a viable antiviral drug candidate. Due to the present inability to grow ferret coronaviruses in culture, further *in vitro* testing has not been possible (57). However, preliminary results against SARS-CoV-2 provide analogous results (58).

While further pathogenesis studies are required to understand FRSCV, disease is undoubtedly multifactorial. In some regions, including North America, it has been demonstrated that genetic variability is relatively low among ferrets (59). Due to centralized breeding, pet ferrets and laboratory ferrets are often sourced from the same location. This lack of genetic diversity could be a risk factor for future coronavirus outbreaks among laboratory or pet ferrets, paralleling previous outbreaks of FCoV in captive cheetahs (60); it has been hypothesized that the low genetic variability among cheetahs, including major histocompatibility complex (MHC) homogeneity, was a factor leading to these devastating outbreaks (61, 62).

## FERRETS AS MODELS FOR HUMAN CORONAVIRUS

**FIG 2:**
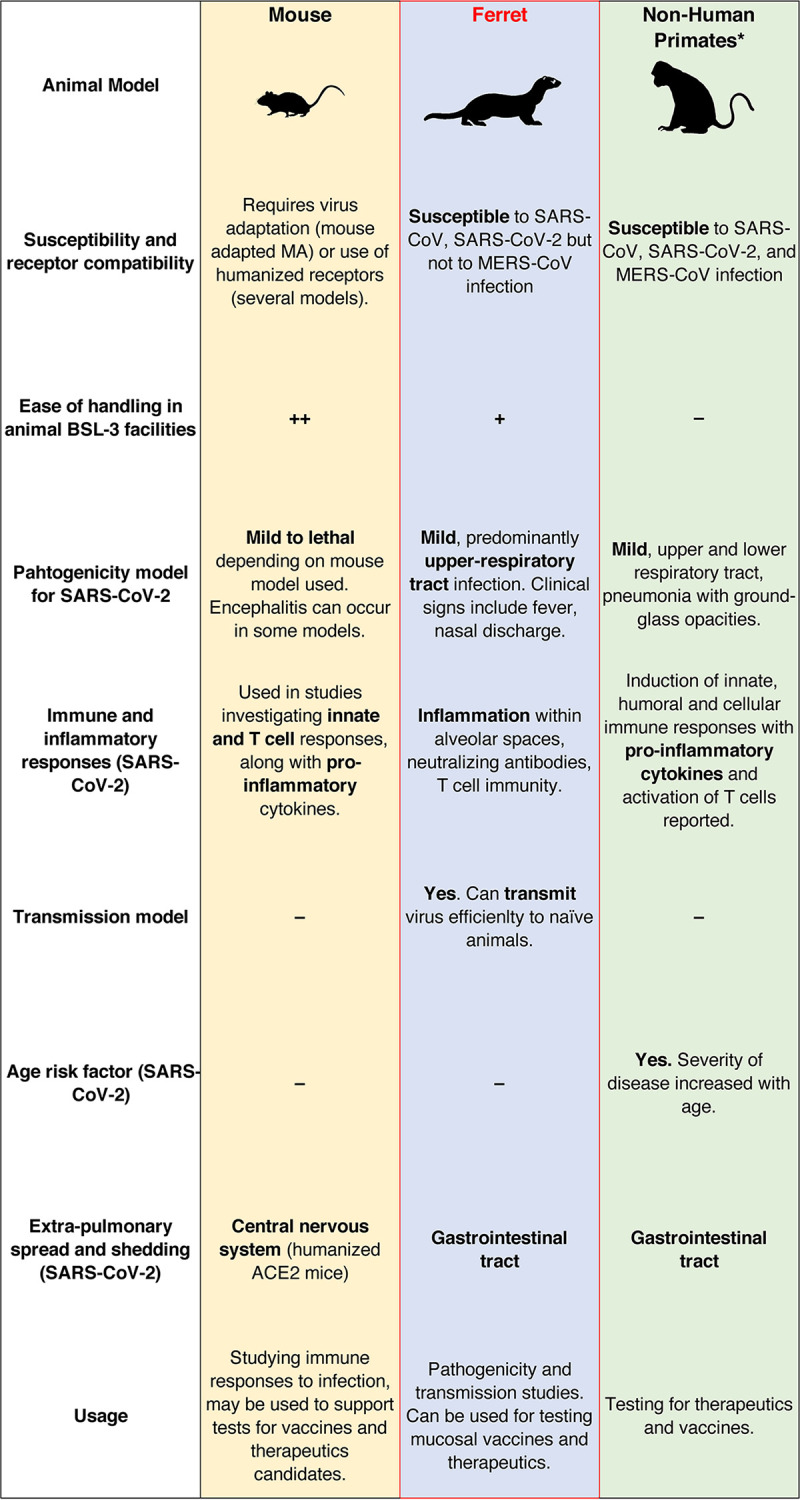
Comparison of mouse, ferret, and nonhuman primates as animal models to study highly pathogenic human coronaviruses. The comparative analysis is based mostly on studies on SARS-CoV-2 infection; however, some parameters are applicable to other human coronaviruses such as SARS-CoV and MERS-CoV. The asterisk denotes that nonhuman primates (NHPs) comprises macaques and African green monkeys.

Ferrets have previously been used as a model for other viruses of human consequence, including influenza virus and respiratory syncytial virus (2, 3). In some regards, ferrets bridge the gap between mouse models and nonhuman primate models, being naturally susceptible to several coronaviruses yet easier to house and maintain compared to nonhuman primates (Fig. 2). Additionally, as a common household pet, the potential role of zoonotic transmission between humans and ferrets remains a possibility. Ferrets are considered susceptible to SARS-CoV and SARS-CoV-2 in laboratory models, with similarities in the ACE2 receptor across humans and ferrets being previously demonstrated (4, 7, 63). Ferrets, however, are not a suitable model of MERS-CoV (Fig. 2). The potential for disease caused by the other human coronaviruses (HCoV-229E, HCoV-NL63, HCoV-OC43, and HCoV-HKU1), in addition to coinfections in ferrets, remains largely unexplored, although one report investigating ferret coronaviruses has noted that HCoV-NL-63 was not amplified in a sample of ferrets in the Netherlands (32).

### (i) SARS-CoV.

Initial investigation regarding SARS-CoV animal models demonstrated that ferrets were a suitable host for the virus and could develop clinical signs (4). In ferrets infected intranasally with the SARS-CoV TOR2 strain (Fig. 1), a common clinical sign was sneezing and in some animals, diarrhea (64, 65), while ferrets inoculated intratracheally with the HKU 39849 strain of SARS-CoV (Fig. 1), developed lethargy at 2 days postinoculation and one ferret, of four, died at day 4 postinoculation (66). However, discrepancies exist in regard to the development of clinical signs, with one study noting no obvious signs, despite viral detection, following challenge with 10^6^ PFU of the TOR2 strain (67). Interestingly, in lung tissue from ferrets inoculated with SARS-CoV TOR2 strain, viral load peaks at approximately day 5 or 6, and although titers decreased, a very small peak in viral titer was observed at approximately 1 month postinfection (64, 65). On reinfection at approximately 1 month after initial infection, viral titers in lung tissue were raised only minimally above baseline, or in the case of the nasal turbinates and pharyngeal fluid, frequently remained below baseline (64, 65). Lung pathology of ferrets infected with SARS-CoV TOR2 has included lymphohistiocytic bronchointerstitial pneumonia, type II pneumocyte hyperplasia, and cellular infiltrates (65). Lung consolidation was evident in all of the ferrets inoculated with HKU 39849 strain of SARS-CoV; one ferret also had an enlarged mesenteric lymph node, and another had a friable liver and mottled spleen, which was similar to the distribution of histologic lesions (66). Histologically, all of the ferrets inoculated with HKU 39849 strain of SARS-CoV displayed diffuse alveolar damage, hepatic lipidosis, and mild lymphoid hyperplasia in the spleen and trachea-bronchial lymph nodes (66). SARS-CoV antigen was present in type II pneumocytes and less frequently in type I pneumocytes or alveolar macrophages, similarly following the pattern of ACE2 expression, which was most common in type II pneumocytes and serous epithelial cells of the trachea-bronchial submucosal glands (66). Outside of the respiratory tract, ACE2 expression has also been demonstrated by RT-PCR in the heart, kidney, and small intestine of the ferret (63). Despite this expression, less is known about SARS-CoV pathogenesis in the heart and kidney. Nonetheless, ferret ACE2 is a suitable receptor for SARS-CoV, with HeLa cells expressing ferret ACE2 being permissive (63). In regards to common gene expression patterns on infection, Cameron and colleagues demonstrated expression of interleukin 6 (IL-6) signaling/complement genes and interferon response genes; however, reinfection resulted in decreased interferon response gene expression (64). CXC chemokine ligand 10 (CXCL-10), a chemokine important for Th1 polarization and potentially involved in the development of acute respiratory distress syndrome (ARDS), has been shown to be elevated both in SARS patients and ferrets infected with SARS-CoV (68). Infection of ferrets with SARS-CoV, after giving alpha2b interferon (IFN-α2b), indicated a predominant role of STAT1 in regard to the immune response (69). In STAT1 knockout mice, infection of SARS-CoV results in severe disease (70).

Ferrets have additionally been utilized as a model to compare vaccination options, including whole killed virus (TOR2 strain), adenovirus vaccines expressing the N and S proteins, and a modified vaccinia Ankara vaccine expressing S or N proteins (67, 71–74). In those administered an adenovirus virus vaccine expressing N and S proteins, clinical signs developed across the experimental groups, despite vaccination; however interestingly, eosinophilic infiltration was not observed across infection groups and may be a limit of the ferret model (73). Similarly, in ferrets administered an adenovirus vaccine with only S protein, mild lung pathology developed following infection, although a T cell response was evident (72). In ferrets vaccinated with formalin-inactivated SARS-CoV and then challenged with SARS-CoV Urbani strain, the vaccination was relatively safe, resulted in quicker clearance of the virus from pharyngeal swabs and a neutralizing antibody response after challenge (71). In a modified vaccinia Ankara vaccine expressing either N or S, vaccination with the S protein followed by viral challenge resulted in hepatitis, potentially mediated by antibody-dependent enhancement (67, 74). In regards to other immunoprophylaxis options, a human monoclonal antibody against SARS-CoV demonstrated efficacy in a ferret model (75).

### (ii) SARS-CoV-2.

The COVID-19 pandemic has necessitated investigation of SARS-CoV-2 infection in the commonly used ferret model. In initial experimental infections with the F13-E and CTan-H strains, viral RNA could be found in the nasal turbinates, nasal washes, soft palate, tonsils, and in rectal swabs, but not in lung tissue, though pathological changes were evident, including vasculitis and type II pneumocyte hyperplasia (76). Clinical signs, namely, fever and loss of appetite were reported but were not widespread among infected animals (76). Seroconversion was evident in this model (76). While an additional study demonstrated the development of clinical signs (fever, lethargy, coughing) (7) in a separate study cohort of only female ferrets, clinical signs were not apparent (9). Additional sources of virus shedding in saliva and urine have also been observed, and the presence of virus in the lungs has been confirmed (7). The potential for direct and indirect transmission after initial ferrets are inoculated via the intranasal route has been demonstrated (7, 8). By day 2 postinoculation, ferrets with direct contact to the initially inoculated ferrets were positive for viral RNA in nasal washes, saliva, urine, and feces and remained positive through day 8, with the exception of urine that remained positive only through day 4 (7). In the ferrets that had indirect contact, nasal washes were positive for viral RNA from day 4 through 8, and fecal shedding was noted on days 4 and 6 (7).

Despite experimental models demonstrating the potential for the development of clinical signs and viral replication, evidence of natural infection in owned ferrets remains unclear. In a household with 29 ferrets exposed to a known COVID-19 case and another person with symptoms, viral RNA was not detectable in samples from the ferrets, while serology did not provide evidence of previous infection (77). Thus, virus and host genetic barriers may help mitigate naturally occurring infections in pet ferrets (77). Despite this single study not demonstrating natural transmission of SARS-CoV-2 from owner to pet, further investigation across additional scenarios remains warranted.

Like SARS-CoV, ferrets have been utilized in exploring vaccine efficacy. In a study of intramuscular and mucosal administered adenovirus type 5-based COVID-19 vaccination (Ad5-nCoV), S-specific serum IgG antibodies and neutralizing antibodies were detected in all vaccinated groups but not in the control group; cellular responses were detected commonly in ferrets in the intramuscular group (5/6) and less commonly in the mucosal group (3/6) (78). Virus load was reduced in nasal washes of the intramuscular group compared to the control group, while no virus was detected in the nasal washes of the mucosal group (78). Last, ferrets have been utilized to investigate potential drug therapeutics for SARS-CoV-2. While the administration of the drugs, lopinavir-ritonavir, hydroxycloroquine sulfate, and emtricitabine-tenofovir showed little impact, the ferret model may hold the potential for future experimental work (79). For instance, with vasculitis reported in some ferrets following infection, this model may provide an accurate representation of therapeutics in human patients (76). Similarly, ferrets may be able to recapitulate long-term disease and shed light on the questions that arise in asymptomatic patients. Further probing of the lack of clinical signs in a cohort of female ferrets may also help provide insight into the sex disparities in human patients (9). Last, with obese individuals being considered at higher risk for COVID-19, the use of a previously described obese ferret model used in influenza research may provide additional insight (80, 81).

### (iii) MERS-CoV.

In 2012, Middle East respiratory coronavirus emerged as a human pathogen (82, 83). In human infection, Middle East respiratory virus (MERS-CoV) uses the host cell receptor dipeptidyl peptidase 4 (DPP4), of which ferret DPP4 is not conducive as a MERS-CoV receptor (84, 85). In addition to being highly divergent compared to human DPP4, glycosylation is different across the two proteins (85, 86). Ferrets have been unsuccessfully investigated as a model for MERS-CoV infection, demonstrating no seroconversion or viral shedding in respiratory swabs following intranasal or intratracheal inoculation (85), even though immunohistochemistry demonstrated DPP4 expression in lung and kidney tissues (87). In the lung, the highest expression of DPP4 included bronchiolar smooth muscle and axonal cells, while no expression was observed in alveolar macrophages, alveolar interstitium, and mesothelium (87). In the kidney, the highest expression of DPP4 occurred in cortical apicular proximal tubular epithelium and axonal cells, while no expression was observed in endothelial cells (87). Though a primary ferret kidney cell line has been previously developed and demonstrated to express DPP4, viral replication has not been observed (87). However, the expression of human DPP4 in primary ferret kidney cells can lead to viral replication (87). Expression of ferret DPP4 on HEK293T cells subsequently infected with recombinant MERS-CoV that expresses tomato red fluorescent protein instead of the ORF5 protein (rMERS-CoV-RFP) did not significantly alter cell infectivity compared to those not expressing ferret DPP4, though the amino acid sequence identity to human DPP4 is nearly 88% (88). Likewise, the expression of fDPP4 in MDCK cells did not allow for rMERS-CoV infection (85).

## CORONAVIRUSES AND OTHER MEMBERS OF THE MUSTELOIDEA SUPERFAMILY

Considering the zoonotic nature of coronaviruses, it is important to contextualize the risk across related *Musteloidea* species, especially given the numerous habitats these species may be found in, including aquatic environments. In 1976, cases of possible FIP-like disease were reported in two captive short-clawed otters (Aonyx cinereus) approximately 7 months after acquisition (89). The first animal succumbed to sudden death and on necropsy appeared anemic and icteric and had large amount of abdominal fluid present (89). The second animal demonstrated neurological signs in addition to the presence of abdominal fluid (89). Across the two cases, pathology was noted in the liver, kidneys, lungs, and mesenteric lymph nodes (89). Viral isolation was attempted but was not successful; however, intraperitoneal inoculation of abdominal fluid from the second case into a domestic cat resulted in fever, weight loss, abortion, and hepatitis (89). While it is not surprising that virus could not be isolated, giving the difficulties to date of doing so, it remains unknown whether this was definitively a manifestation of a coronavirus infection, and additionally, it was not apparent how these animals may have become infected. Shortly after these cases, in 1979, Horzinek and Osterhaus, were unable to find coronavirus antibody in a single tayra (Eira barbara) (90). Further studies have investigated coronavirus exposure or carriage across numerous species of the *Mustelidae* family (Table 1). In addition to these studies to investigate natural coronavirus exposures in wild mustelids, continued surveillance is optimal, including across other geographic regions. For example, a novel coronavirus has previously been detected in a Chinese ferret badger (Melogale moschata*)* in southern China (91).

**TABLE 1:** Coronavirus surveillance in mustelidae species

Species	Location	Yr	Test objective^*a*^	Result (no. positive/total no. tested)	Reference
Tayra (*Eira barbara*)	Not given	1979	IFT against TGEV	0/1	90
American badger (Taxidea taxus)	British Columbia	1996–2001	CCoV antibodies	0/7	107
Fisher (Pekania pennant, formerly Martes pennant)	British Columbia	1996–2001	CCoV antibodies	4/28	107
Wolverine (Gulo gulo)	British Columbia	1996–2001	CCoV antibodies	0/20	107
Eurasian otter (Lutra lutra)	Portugal	1995–2011	CCoV RT-PCR	1/1	108
North American river otter (Lutra canadensis)	New York, USA	1996	CCoV or FCoV antibodies	0/38	109
Stone marten (Martes foina)	Portugal	1995–2011	CCoV RT-PCR	0/3	108
	Portugal	2008–2011	7b gene RT-PCR	0/1	110
Pine marten (Martes martes)	Portugal	1995–2011	CCoV RT-PCR	0/1	108
European badger (Meles meles)	Portugal	1995–2011	CCoV RT-PCR	0/1	108
	Portugal	2008–2011	7b gene RT-PCR	0/1	110

a IFT, indirect immunofluorescence test.

The endangered red panda is the extant species of the family *Ailuridae* of the *Musteloidea* superfamily (92). Among captive red pandas, antibodies against canine coronavirus are relatively rare, including in those that have been vaccinated (93, 94). The lack of CCoV antibodies may provide evidence of limited exposure to the virus; however, it additionally raises questions in regard to apparent vaccine failures and future protection. Interestingly, in an available abstract from Qin and colleagues, it appears that CCoV antigen has been detected in fecal samples from red pandas (95).

The *Procyonidae* family includes the North American raccoon (Procyon lotor), coatimundis (*Nasua* sp.), kinkajous (Potos flavus), and other species (96). In regards to these species and coronaviruses, the literature to date has focused on the raccoon. The first case report of a coronavirus causing disease was of a juvenile moribund raccoon in Colorado (USA), which was also found to be shedding cryptosporidia and parvovirus (97). In addition to upper respiratory signs and diarrhea, necropsy revealed gastroenteritis, bronchopneumonia, and intestinal blunting with neutrophil infiltration (97). The multipathogen infection of this single animal eliminates the ability to draw definitive conclusions about coronavirus infections and pathogenesis in raccoons; however, in environments where raccoons and domestic species might be interacting, it may be possible to spread infection between these species. In a study of 379 feral raccoons in Japan, an antibody response against TGEV was detected in 11 samples, though many of these titers were low (98). However, among these 11 samples, an elevated titer against TGEV frequently corresponded with an elevated titer against CCoV (98). Antibodies against bovine coronavirus (BCoV) or porcine epidemic diarrhea virus (PEDV) were not evident in these animals (98). In a separate serological study of Japanese raccoons, 7 out of 100 animals were noted to have positive antibody titers against CCoV (99). Positive antibody responses in novel species may represent cross-species transmission of coronaviruses and may indicate the larger disease ecology, including the potential for recombination events. Additionally, it is not highly likely that previous exposure may provide cross protection in these species.

## CONCLUSIONS

The consideration of coronaviruses in the *Musteloidea* superfamily provides insight into natural coronavirus infections, potential avenues for laboratory investigation, and considerations for conservation medicine. Additionally, whether considering these species from the perspective of pet ownership, farm-raised animals, or wildlife, the potential for reverse zoonotic transmission currently remains a threat. Further, the potential for dual infections of a species-specific coronavirus and SARS-CoV-2 may result in novel disease progressions. Last, a major challenge inhibiting further exploration includes challenges with viral isolation (16, 40). The inability to propagate these viruses creates barriers for understanding basic virology, as well as making progress toward prevention or treatment strategies. Recent work with FCoV has indicated that engineering cells to overexpress receptors, and activating proteases, or to modulate interferon signaling responses can improve cell culture of certain strains (100, 101), and this approach combined with selection of cell culture-adapted strains is likely to improve virus isolation for this understudied but potentially highly important niche of the *Coronaviridae*.

While there is no evidence for minks or other musteloidea as intermediate hosts for the zoonotic transfer of SARS-CoV-2 from bats to humans, the fact that minks have now been clearly identified as hosts for SARS-CoV-2 does raise the issue of this species as intermediate hosts for the emergence of further COVID-19 outbreaks in humans. Also, coronaviruses are highly recombinogenic (102), and there is the risk of coinfections between MCoV and SARS-CoV-2 generating novel viruses. While there is little precedent for recombination across the alpha- and betacoronaviruses, this cannot be excluded. Swine acute diarrhea syndrome coronavirus (SADS-CoV) is a highly pathogenic swine coronavirus that was identified in China in 2018 and is closely related to a bat virus, BatCoV-HKU2 (103, 104). Based on replicase gene phylogenetic analysis, BatCoV-HKU2 was assigned to the alphacoronavirus genus (105). However, further phylogenetic and structural analyses revealed that the spike proteins of both BatCoV-HKU2 and SADS-CoV bear more resemblance to betacoronavirus spike proteins, suggestive of a recombination event involving ancestral alpha- and betacoronaviruses (103, 106). These findings along with the studies covered in this minireview warrant increasing surveillance efforts to monitor circulating coronaviruses in both wild and domesticated members of the *Musteloidea*.
